# Policy implementation and recommendations to address the double burden of malnutrition in South Africa: expert assessment using the expanded Healthy Food Environment Policy Index (Food-EPI)

**DOI:** 10.1186/s12916-025-04191-y

**Published:** 2025-07-01

**Authors:** Nicole Holliday, Peter Delobelle, Carmen Klinger, Zandile June-Rose Mchiza, Olufunke Alaba, Stefanie Vandevijvere, Peter von Philipsborn, Karin Geffert

**Affiliations:** 1 Institute for Medical Information Processing, Biometry and Epidemiology (IBE), Chair of Public Health and Health Services Research, Faculty of Medicine, LMU Munich, Marchioninistrasse 15, Munich, 81377 Germany; 2 Pettenkofer School of Public Health, Elisabeth-Winterhalter Weg 6, Munich, 81377 Germany; 3 Chronic Diseases Initiative for Africa, University of Cape Town, Groote Schuur Hospital, Observatory 7925, J47/86 Old Main Building, Cape Town, South Africa; 4 Department of Public Health, Vrije Universiteit Brussel, Laarbeeklaan 101, Brussels, 1090 Belgium; 5 Non-Communicable Disease Research Unit, South African Medical Research Council, Francie Van Zijl Drive, Parowvallei, PO Box 19070, Tygerberg, 7505 South Africa; 6 School of Public Health, University of the Western Cape, Robert Sobukwe Rd, Bellville, Cape Town, 7535 South Africa; 7 Health Economics Unit, School of Public Health, University of Cape Town, Observatory, Cape Town, 7925 South Africa; 8 Department of Epidemiology and Public Health, Sciensano, Rue Juliette Wytsmanstraat 14, Brussels, 1050 Belgium

**Keywords:** Food-EPI, Food environments, South Africa, Double burden of malnutrition, Policy recommendations, Public health policies

## Abstract

**Background:**

South Africa faces a double burden of malnutrition (DBM), the coexistence and interaction of multiple forms of malnutrition (undernutrition, micronutrient deficiencies, and overweight/obesity) within individuals and households and across the life course. A healthy food environment is necessary to reduce this DBM. The Healthy Food Environment Policy Index (Food-EPI) can be used to evaluate the implementation of public nutrition and food environment policies in comparison with international best practices. The aim of this study was to assess the extent of implementation of healthy food environment policies in South Africa using an expanded DBM Food-EPI framework, benchmark policies against international best practices, develop priority policy recommendations, and compare implementation progress since the 2016 South African Food-EPI assessment.

**Methods:**

From October 2023 to March 2024, a panel of 23 national experts from different tiers of government (Department of Health), academia, and civil society was invited to participate in the Food-EPI assessment. Through two workshops and online feedback, experts evaluated the implementation of food environment policies across 60 indicators, compared these policies to international best practices, and proposed and prioritized a list of policy actions based on perceived implementation gaps.

**Results:**

Of the 23 invited experts, 13 participated in the benchmarking workshop in which about 70% of indicators were rated at very low to low levels of implementation. Overall, of the 48 original indicators, the mean level of implementation improved from 2016 to 2024. Of the 12 indicators that addressed the DBM, eight were rated at very low to low levels of implementation. The experts (original panel plus four additional participants) then proposed ten priority actions, mainly across the domains of Food Promotion, Food Prices, Funding, and Platforms for Interaction.

**Conclusions:**

Application of the expanded Food-EPI in South Africa showed improvements for the original indicators compared with 2016 and highlights the need for additional policy efforts to improve public nutrition policy and address the DBM.

**Supplementary Information:**

The online version contains supplementary material available at 10.1186/s12916-025-04191-y.

## Background

Despite having a positive food balance (i.e., producing sufficient food to feed its population), South Africa still faces a significant burden of undernutrition and micronutrient deficiencies. An estimated 12% of the population experiences chronic hunger [1]. Micronutrient deficiencies are common among young children and adults, with estimates of anemia ranging from 61% among children under 5 years to 31% and 17% of women and men 15 years or older, respectively [1]. The rate of stunting among children under 5 years has remained mostly stagnant for the past 20 years at about 27% [1]. At the same time, South Africa has seen a dramatic rise in the prevalence of overweight and obesity in the last decades and now has one of the highest rates in Africa. Overweight or obesity is present in nearly 68% of adult women, 31% of adult men, and 21% of adolescents (15–19 years) and is linked with diet-related non-communicable diseases (DR-NCDs) that account for 51% of the country’s annual deaths [1–3]. This coexistence of multiple forms of malnutrition (obesity, DR-NCDs, undernutrition, and micronutrient deficiencies) in individuals and populations across the lifespan has been named the double burden of malnutrition (DBM) [4].

The DBM is in part driven by the food environment in South Africa, which consists of the interface between consumers and the social, economic, physical, political, and cultural elements of the food system that shape individual diets and nutritional status [5, 6]. Food environment domains typically include factors such as food affordability, availability, accessibility, quality, sustainability, and marketing, which in South Africa have changed significantly over the past 50 years [7, 8]. A marked increase in the availability and sales of ultra-processed foods has been noted, including foods with high sodium, saturated fat, and sugar content, in both urban and rural areas [8]. For example, between 2005 and 2010, the sales of packaged foods such as snack bars, ready meals, and noodles increased by more than 40% [9]. The 2016 South African Demographic and Health Survey (SADHS) found that of a nationally representative sample of over 10,000 respondents across the nine provinces, 40% of men and 32% of women consumed fried foods at least once a week, and 18% of men and 17% of women consumed fast foods at least once a week [3]. Food advertising plays a significant role in these changing consumption patterns, and studies have found that persuasive marketing across media (e.g., TV, billboards, social media) and outdoor advertising disproportionately advertise unhealthy foods, sugar-sweetened beverages (SSBs), alcoholic beverages, and breastmilk substitutes, despite regulations, industry pledges, and WHO recommendations to the contrary [1, 10–12].

Comprehensive policy actions are necessary to create healthier food environments and reduce the DBM. To achieve this goal, it is important to take stock of government policies, including their extent of development and implementation, and compare this to international best practice. The Healthy Food Environment Policy Index (Food-EPI) is the current gold standard for evaluating the extent of implementation of healthy food environment policies by governments compared to international best practice [13]. Created in 2013 by the International Network for Food and Obesity/NCDs Research, Monitoring and Action Support (INFORMAS) to evaluate food environment policy and action related to the prevention of overweight, obesity, and DR-NCDs, the tool has been implemented in over 40 countries worldwide. Studies in sub-Saharan Africa, however, indicated that the tool lacks indicators related to undernutrition and the DBM [14]. Hence, in 2020, INFORMAS and experts in food environment research started to adapt the Food-EPI index to include policy areas relevant to the DBM, including breastfeeding, food safety, water, sanitation and hygiene (WASH), and growth monitoring, resulting in the addition of twelve new good practice indicators [14].

Food-EPI was first implemented in South Africa in 2016, in which 11 experts participated, as reported in an 11-country study paper published by Vandevijvere et al. [19]. This first assessment, however, did not consider the DBM, and since then, new nutrition policies have been enacted, including a tax on sugar-sweetened beverages (SSBs) commonly known as the Health Promotion Levy [15]. The aim of this study was hence to engage experts to assess the current extent of implementation of healthy food environment policies in South Africa using an expanded Food-EPI tool with indicators addressing the DBM; to benchmark policies in comparison to international best practice; to develop a prioritized set of policy and infrastructure support recommendations for government; and to assess progress for the original indicators compared with the 2016 Food-EPI assessment.

## Methods

### Description of Food-EPI tool

The Food-EPI tool originally developed by Swinburn et al. [13] is divided into two components, “Policies” and “Infrastructure Support,” which consist of seven and six domains, respectively, each comprising a number of good practice indicators. “Policies” include those components that “address the key aspects of food environments that can be influenced by governments” while “Infrastructure Support” includes components that “facilitate policy development and implementation” [13].

### Food-EPI process

The South Africa Food-EPI 2023/2024 assessment was conducted in eight steps: (1) adaptation of the tool to the South African context, including updating the indicators related to the DBM; (2) a comprehensive review of the implementation of national food and nutrition policy; (3) verification of the collected public policy evidence by project stakeholders from academia and government; (4) identification of international best practice examples (benchmarks) for good practice indicators; (5) benchmarking workshop with experts to assess the extent of implementation of public policy compared to international best practice; (6) identification of policy actions for future implementation; (7) priority setting workshop to review and update the list of recommended policy actions; and (8) online rating of recommended actions and consensus on the final list of suggested priority actions.

#### Adaptation of tool to South African context

**Fig. 1:**
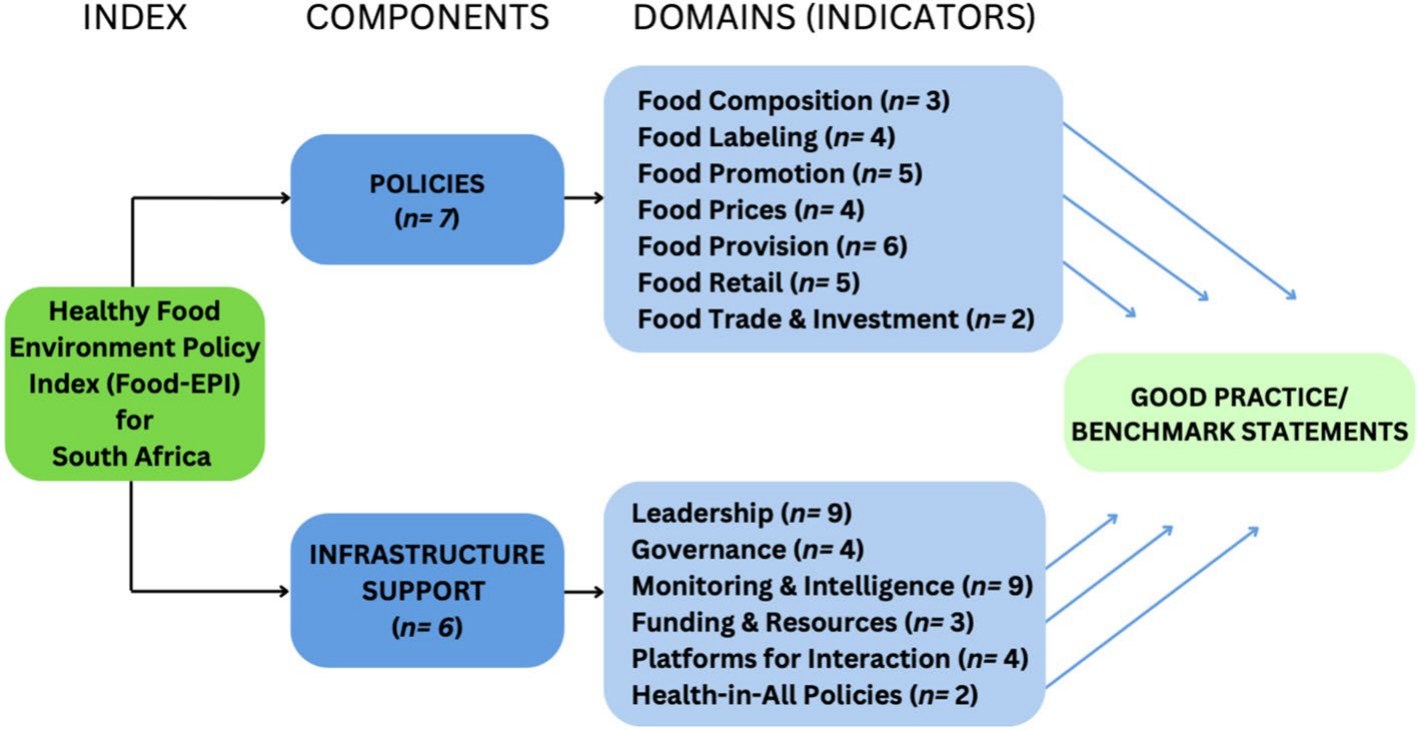
Expanded Food-EPI framework for South Africa (adapted from Swinburn et al. [13])

The components, domains, and indicators of the Food-EPI tool, as well as additions for newly formulated DBM indicators from the Food-EPI assessments in Tanzania and Uganda [16, 17] were carefully reviewed through consultation with experts in food system and nutrition policy research in South Africa. The final tool included 29 indicators within the Policy component and 31 indicators within the Infrastructure Support component (Fig. 1). This resulted in an expanded Food-EPI tool of 60 indicators, including twelve new indicators developed by INFORMAS in 2020 to better assess the implementation of policies addressing the DBM.

#### Comprehensive review of food environment related public policies in South Africa

Evidence on implementation for each of the 60 good practice policy and infrastructure support indicators was gathered during an online search from May to September 2022. Evidence collected during the 2016 Food-EPI assessment was used as a basis and checked for continued relevance, complemented by new evidence on changes or updates in policy and implementation. The searched databases included government websites, non-governmental organizations’ (NGO) publications and their websites, academic literature and websites, and news articles. Data were compiled in an evidence document with policies sorted into their relevant indicators and checked for internal validity by the study investigators.

#### Validation of evidence by stakeholders

The updated evidence document was shared with key project stakeholders (two food and nutrition experts; one legal expert) and a representative of the National Department of Health to receive feedback on the accuracy and completeness of the evidence.

#### Identification of international best practice examples (benchmarks)

The list of international best practice examples (i.e., benchmarks) was informed by best practice examples used in other Food-EPI assessments in Africa (e.g., Ghana, see [24]) and a list of best practice examples used for the newly formulated DBM indicators in the Food-EPI assessment in Tanzania and Uganda [16, 17]. All best practice examples were checked for their relevance.

#### First workshop: benchmarking current policies

Through stakeholder mapping, we identified experts to invite to participate in the expert panel. To gather perspectives from different sectors and levels, 23 experts from different tiers of government (Department of Health), academia, and civil society were invited to participate in a workshop to assess the extent of implementation of food and nutrition policy in South Africa.

Members of the expert panel received a copy of the evidence document to review prior to the workshop. During the workshop, the evidence and benchmarks were discussed for each indicator. Experts discussed discrepancies in the evidence document and provided their views on the extent of policy implementation. The workshop lasted half a day and was conducted in a hybrid format to optimize participation. At conclusion, experts received a link including an individual access key to an online survey platform (LamaPoll, 2024), which allowed participants to evaluate the extent of policy implementation for all indicators compared with international best practice using a five-point Likert scale, as recommended in the Food-EPI protocol (1 = less than 20% implementation compared to best practice, 2 = 20–40%, 3 = 40–60%, 4 = 60–80%, 5 = 80–100%) [18]. The survey allowed users to select “no answer” when they felt unable to provide an answer, and space was available for written comments after each indicator.

#### Initial drafting of recommended policy actions

Upon analysis of the results from the benchmarking workshop, the core research team drafted an initial list of recommended actions based on indicators for which implementation was rated at less than 20%. Recommended actions were framed in line with those developed in Food-EPI assessments from other African countries (e.g., Ghana, Senegal) and resulted in a list of 17 recommended actions (11 policy, 6 infrastructure support) that was sent for revision to the wider research team. The latter included academic research experts in public health nutrition and food policy who were familiar with the South African context.

#### Second workshop: prioritizing actions

The 13 experts from the first workshop, along with four additional/newly identified experts in public health nutrition and food policy, were invited via email to participate in a workshop on the prioritization of policy actions. They received a copy of the list of recommended actions and an updated version of the evidence document based on discussions in the first workshop. During this second workshop, which was hosted in a hybrid format in March 2024, results from the first benchmarking workshop were shared to provide an overview of policy and infrastructure support areas that were rated higher or lower in terms of implementation. The initial list of recommended actions was discussed amongst the expert panel, and policies were added, removed, or edited as necessary.

#### Online rating exercise to prioritize policy actions

After the workshop, members of the expert panel were asked to rate the 17 actions along the two dimensions of importance and achievability, based on the criteria of need, impact, equity, and other positive/negative effects; and feasibility, acceptability, affordability, and efficiency, as per the Food-EPI protocol (Table 1) [18].

**Table 1:** Ranking criteria for policy priority setting

Importance	Achievability
Need The size of the implementation gap	Feasibility How easy or hard the action is to implement
Impact The effectiveness of the action on improving food environments and diets (including reach and effect size)	Acceptability The level of support from key stakeholders including government, the public, public health, and industry
Equity Progressive/regressive effects on reducing food/diet-related health inequalities	Affordability The cost of implementing the action
Other positive effects (e.g., on protecting the rights of children and consumers)	Efficiency The cost-effectiveness of the action
Other negative effects (e.g., regressive effects on household income, infringement of personal liberties)	

For the 11 Policy actions, a total of 22 points could be allocated to each dimension with no more than five points per action (divided between the dimensions of importance and achievability). For the six Infrastructure Support actions, twelve points could be allocated to each dimension with no more than five points allowed per action. Experts were given the option to either complete the rating using a web-based survey platform (PollUnit, 2024) or an Excel worksheet. Due to technical difficulties with the survey, the online option was abandoned, and all results were shared in Excel with the research team.

### Data analysis

Data were analyzed using Microsoft Excel. For the prioritization exercise, for each criterion, we calculated the arithmetic mean of the points assigned to each recommendation by participating experts. As a next step, a summary score was calculated for each recommendation by summing the scores for each criterion to obtain an overall ranking of recommendations. The rankings were plotted on a graph with achievability on the *y*-axis and importance on the *x*-axis, and those actions within the top right quadrant (≥ 10 points on achievability and ≥ 15 points on importance) were included in the final list of recommended actions (as described in the Food-EPI protocol) [18].

For comparison of indicators included in the 2016 data collection, we subtracted the arithmetic mean of the 2016 rating from the arithmetic mean of the 2023/4 rating. Data from the 2016 assessment were provided by the original authors as they were not publicly available. If no indicator was available, the difference was not calculated. We considered any difference in means of at least one (1.0) as a change in level of implementation.

### Ethical considerations

This research received approval from the ethics review board of the University of Cape Town (567/2022), the University of the Western Cape (ref number BM22/2/7), and Ludwig-Maximilians-Universität München (LMU Munich) (ref number 22–0505). Participants were informed of their rights and requested to provide written consent prior to their participation in the workshops.

## Results

### Composition of the expert panel

Of the 23 original invitees, 13 agreed to participate (11 academics, 1 civil society, 1 government) and attended the benchmarking workshop. The second, priority-setting workshop was attended by the same 13 experts and by four additional experts from academia. From this group, ten experts completed and returned the Excel sheet on the ranking of priority actions.

### Level of implementation of policy and infrastructure support—benchmarking results

Table 2 presents the benchmarking results. Regarding the implementation of each indicator against existing international best practice, 20 indicators were rated as “very low, if any” (< 20%), 22 as “low” (20–40%), 13 as “medium” (40–60%) and five as “high” (60–80%) implementation. No indicator was rated as “very high” implementation (80–100%).

**Table 2:**
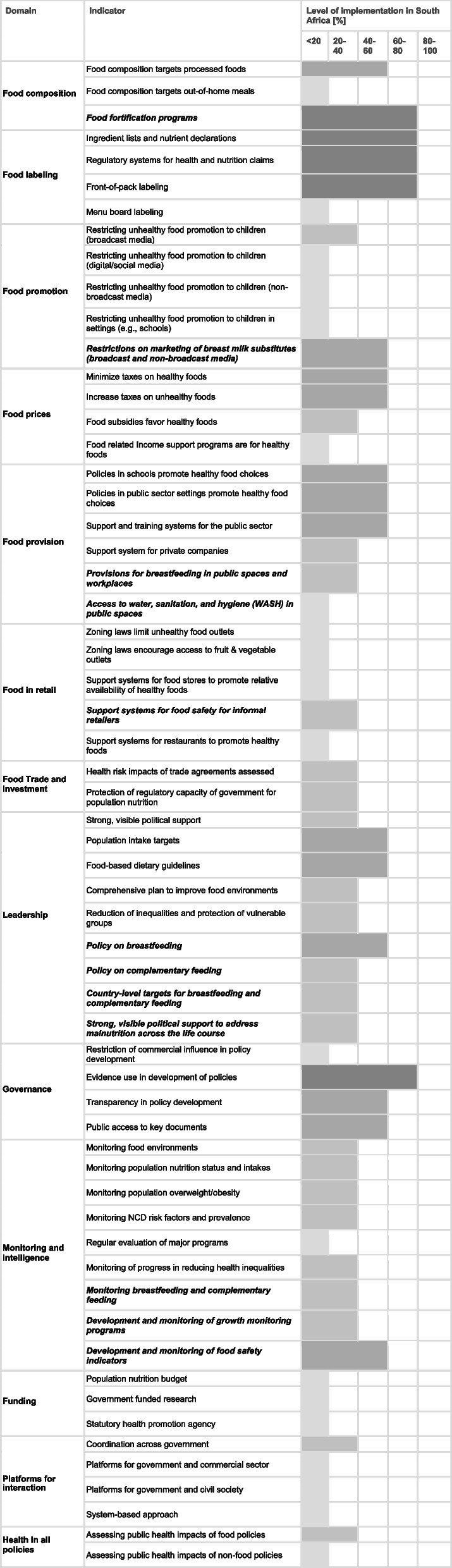
Benchmarking results of level of implementation of good practice indicators in South Africa (2024)

Bolded and italicized indicator text reflects the indicators added to address the DBM. Shading reflects levels of implementation with lighter shading indicating lower levels of implementation and darker shading indicating higher levels of implementation

Indicators within the Food Policy component were generally rated higher than indicators within the Infrastructure Support component. Within the Food Policy component, the most highly rated domains were Food Labeling, Food Provision, and Food Composition. In the Infrastructure Support component, the indicator “Evidence use in the development of policy” in the Governance domain was the most highly rated.

The indicators for which implementation was rated lowest were roughly equally divided between the components of Food Policy and Infrastructure Support (11 and 9 indicators, respectively). The domains that rated lowest were Food in Retail, in particular zoning laws, Food Promotion, Funding, Platforms for Interaction, and Health in All Policies.

Of the twelve additional DBM indicators, one was rated as “high” implementation, three were rated as “medium,” seven were rated as “low,” and one was rated as “very low.” Food fortification programs were rated as having high implementation, whereas the lowest rated implementation was on access to WASH (water, sanitation, and hygiene) in public areas.

### Ranking of priority actions

**Fig. 2:**
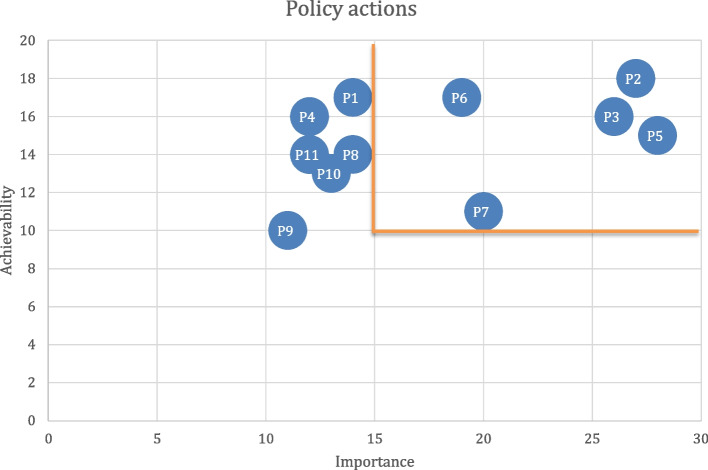
Rating of priority actions within Policy component. Bolded lines indicate the cut-off thresholds for Achievability and Importance

**Fig. 3:**
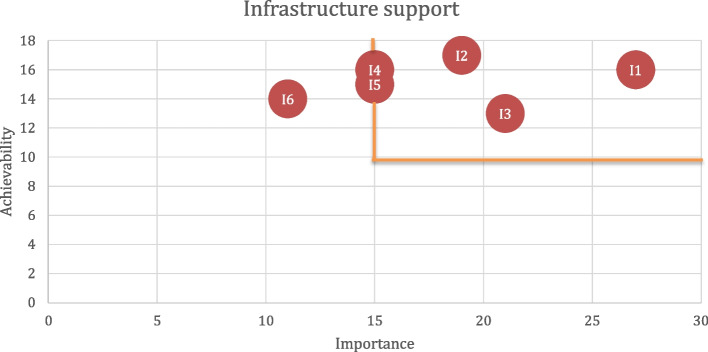
Rating of priority actions within Infrastructure Support component. Bolded lines indicate the cut-off thresholds for Achievability and Importance. Note: I4 and I5 are included within the threshold as they received 15 points on Importance

Figures 2 and 3 present the results from the rating exercise to identify priority actions under Policy actions and Infrastructure Support actions.

Of the 17 proposed actions during the workshop (available in the supplementary material), ten actions were prioritized, five within Policy Action and five within Infrastructure Support (Table 3).

**Table 3:** Prioritized policy and infrastructure support actions

Domain	Food Policy Actions
Food Promotion	P2: Pass a legislation to regulate the promotion, sponsorship and advertisement of unhealthy food and drinks (with sugar, and other nutrients of concern (saturated fatty acids, salt)) in the school environment and other settings where children gather, enforceable with fines P3: Enforce legislation to regulate the promotion, sponsorship, advertisement of unhealthy food and drinks (with sugar, and other nutrients of concern (saturated fatty acids, salt)) towards children in print and online/social media as well as other non-broadcast media, enforceable with fines
Food Price	P5: Implement strategies to increase the affordability of healthy foods with a focus on vulnerable populations (e.g., subsidies, removal of fiscal taxes) P6: Implement and/or increase taxes on unhealthy foods that will raise their price, in particular increase SSB taxation to the 20% threshold P7: Implement income support programs for healthy food
Domain	Infrastructure Support Actions
Platforms for Interaction	I1: Develop and implement policies to regulate relationships and influence of commercial industry on government I4: Develop and implement transparent and clearly-mandated platforms for interaction with both civil society and the commercial sector regarding issues of nutrition I5: Strengthen cross-sectoral platforms for coordination of nutrition and nutrition-related policies and plans
Monitoring and Intelligence	I2: Ensure implementation of evaluation of policies and programs on population nutrition in a timeous way to ensure accountability
Funding	I3: Allocate adequate funding for a population nutrition budget, government funded research, and the funding for health promoting agencies

### Comparison with the 2016 assessment

The 2023/2024 assessment was characterized by a response rate of 43–57% (first and second workshop, respectively), including a participant from the national government, compared to a response rate of 28% and no participation from government stakeholders in the 2016 assessment. Eight indicators were rated lower in 2024 than in 2016, primarily in the domains of Leadership and Platforms for Interaction. Most other indicators were rated higher in 2024 compared to 2016. Eight indicators had a difference in mean of at least one point (1.0) between 2016 and 2024, indicating a change in the level of implementation category. Substantial improvements were seen in front-of-pack labeling policies and nutrient and health claims regulations, taxes on unhealthy food, evidence use in policy development, and assessing public health impacts of food policy. Table 4 presents the difference in means for each indicator between the 2016 and 2024 assessment. The twelve indicators for the DBM were not included in the 2016 assessment and were marked NA (DBM indicators bolded and italicized).

**Table 4:**
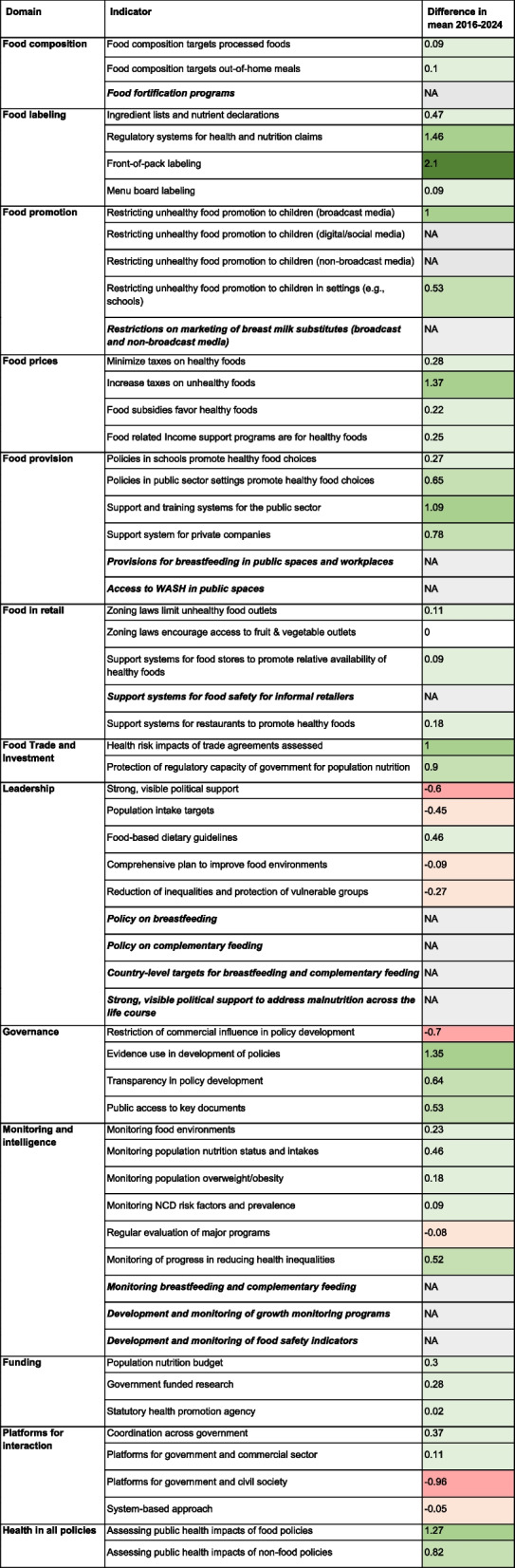
Comparison of indicator ratings between 2016 and 2024

Means are used for calculating the difference between ratings. Color coding: lightest green <0.5, light green 0.5-<1.0, darker green 1.0-<2.0, darkest green ≥2.0 , light red <0.0, dark red <−0.5

## Discussion

In this study, 70% of all Food-EPI indicators for South Africa were rated at a very low to low level of implementation, and 30% were rated as medium to high implementation, which aligns with the results from the 11-country study that included results of the South Africa assessment in 2016 [19]. In another review of four countries in South Asia, not a single country received a strong or very strong rating on the Likert scale of 1–5, and no policies existed for nearly a quarter of the indicators [20]. A pooled assessment of 11 European countries also showed that in only five countries any Policy or Infrastructure Support domain was rated high [21]. In New Zealand, the first country in which Food-EPI was evaluated and has been evaluated three times, 60% of the indicators were rated as low or very low, whereas 15% were rated as high in the latest 2020 assessment [22].

In our study, 13 of the 23 invited experts (57%), including 11 from academia, 1 from civil society, and 1 from government, participated in the benchmarking exercise, and 10 in the prioritization of policy actions (43%), which is higher than participation in the 2016 assessment in South Africa, which had a 28% response rate and included 11 participants (10 academia, 1 civil society). This is roughly equivalent to participation noted in other countries, such as Senegal (50%) [23], Ghana (46%) [24], Ireland (50%) [21], and Chile (46%) [19].

So far, and to the authors’ knowledge, only five countries have used a version of the Food-EPI tool with adaptations for the DBM (Peru, Ethiopia, Burkina Faso, Benin, and Senegal) [14, 23, 25–27]. Of these, two used document analysis with no direct expert involvement (Ethiopia and Burkina Faso) [25, 26], one created new indicators based on Food-EPI and WHO-identified double-duty actions (Peru) [27], and one considered DBM in the prioritization of actions, but not the indicator benchmarking process (Senegal) [23]. Although general comparisons on policy areas of high implementation and those that require strengthening can be made across countries, direct comparisons can only be made with Benin, which applied the same twelve DBM indicators [14].

Implementation levels for DBM indicators were similar between South Africa and Benin. In South Africa, eight DBM indicators (67%) were rated as very low to low, and four (33%) were rated as medium to high (three rated medium and one rated high). In Benin, none were rated as high, but six (50%) were rated as medium [14]. For example, food fortification was rated low in Benin but high in South Africa due to the mandatory food fortification which has been in place in the latter since 2003 [28]. Standards for packaged foods in South Africa are also relatively good, as shown by the compliance with sodium restrictions after implementation of its final targets in 2019 [29]. Leadership-related indicators in Benin, on the other hand, were rated higher than in South Africa, which experts in our panel attributed to the lack of progress on the enactment of national policy (e.g., National Policy on Food and Nutrition Security), as illustrated by the remaining high levels of food insecurity in the country [30].

The highest rated domain in South Africa was Food Labeling, for which all but one indicator was rated as high. The indicators showed a positive change between 2016 and 2024 and reflect not only existing regulations on ingredient lists and nutrient claims, but also the 2023 proposed draft food labeling regulation, which would expand restrictions on misleading claims (e.g., “smart” or “super-food”) and implement mandatory front-of-package warning labels (FOPL) for foods high in sugar, sodium, and saturated fat [31]. Taxes on unhealthy foods, in the Food Prices domain, were also rated higher in 2024, linked to the introduction of the sugar-sweetened beverage tax in 2018. The tax was enacted, however, at a suboptimal level (11%), despite research showing promising results in terms of reduction in consumption when using a 20% tax rate [15].

Food Promotion indicators, on the other hand, were rated low, apart from the restrictions on the marketing of breast milk substitutes, which were regulated in 2012 but need to be enforced and updated. Currently, there are few mandatory policies that address the many dimensions of food marketing; most marketing limitations are voluntary pledges from industry [32]. In 2023, however, a draft White Paper on Audio and Audiovisual Content Services Policy Framework was released by the Department of Communications and Digital Technology, which specifies the need to align regulations that protect children from harmful marketing across all types of media [33]. Regulation is also due for non-broadcast media and, specifically, in the marketing of unhealthy foods in school settings, which currently relies on guidelines from the Department of Basic Education.

Low implementation ratings in the Food in Retail domain included zoning laws limiting unhealthy restaurants and encouraging access to fruit and vegetable outlets, likely reflecting challenges related to the different tiers of governance of food environment policy in South Africa, where the mandate for zoning policies lies within local jurisdictions. In the Food Prices domain, which rated medium, the indicator of income support programs for healthy foods was rated as very low, due to the fact that these support programs (e.g., child support grant) are not directly linked to food support. This constitutes a significant gap in a country facing remaining high levels of poverty and food insecurity as outlined above [34]. Support systems for formal and informal food outlets are also non-existing or skewed towards more affluent population groups.

For indicators within the Infrastructure Support component, a lack of political clout was noted by experts and attributed to inadequate management of conflicts of interest with industry, limited policy coherence and support of different tiers of government, and a low prioritization of the nutrition agenda overall. Very low ratings were noted for indicators on budgetary allocations (including lack of a ring-fenced budget for health promotion), the evaluation and monitoring of nutrition policy, and the establishment of a health promotion agency, which was discussed in view of the rollout of National Health Insurance, but never realized [35]. The domain of Platforms for Interaction also received very low ratings and showed a negative change in two indicators compared with 2016, which was attributed by the expert panel to the siloed approach in policy development and implementation taken by government, limited formal platforms for interaction with civil society, and a lack of structure and guidance for interaction with the private sector.

Based on these ratings, policy actions deemed relevant and achievable focused on legislation to regulate food marketing towards children in all environments; raise the Health Promotion Levy to a 20% threshold; develop income support programs for healthy food; and increase the affordability of healthy food focusing on the most vulnerable populations. The actions should be supported by policies aimed at regulating the relationship with industry, allocation of sufficient funding, and implementation of monitoring and evaluation mechanisms to ensure and increase accountability. Lastly, other recent developments by INFORMAS, such as the expansion of the Food-EPI tool to include sustainability and climate change indicators, could be an important next assessment in South Africa given the direct link between these environmental issues and food security and nutrition in the region [36].

### Strengths and limitations

This study was limited in scope by the relatively low number of participants. The time effort needed rendered it difficult to recruit many participants. Another limitation was the limited participation of government and civil society stakeholders in setting priority actions. However, as noted by Nieto et al. (2019), more inclusive government and NGO participation could also introduce a potential information bias as government employees may be ranking their own performance and NGO participants may evaluate more strongly based on their civic agendas [37].

The primary strength of this study was to provide an updated overview of the extent of food and nutrition policy development and implementation in South Africa compared with the assessment conducted in 2016. The study also included additional indicators relevant to the DBM as well as a list of recommended policy actions which will be shared with local and national stakeholders.

## Conclusions

This assessment of the extent of implementation of food environment related public policy in South Africa using an expanded version of the Food-EPI tool to address the DBM found that some progress has been made in policy areas that promote healthy food environments compared with a previous assessment in 2016, in particular in the domains of Food Labeling and Food Prices. However, significant gaps remain, including in policies that address the DBM. Overall, thirty percent of indicators were rated as medium to high implementation while 37% were rated as low and 30% as very low. Proposed priority actions emphasize the need for legislation limiting unhealthy food marketing to children, supporting programs for healthy food acquisition, and increasing the affordability of healthy food focusing on the most vulnerable populations. The actions should be supported by policies aimed at regulating relationships with industry, sufficient budget allocation, and adequate monitoring and evaluation mechanisms. The recommendations will be shared with stakeholders working in food environment related policy in South Africa using knowledge translation and advocacy efforts. The expanded Food-EPI tool can be used in other settings facing a similarly high DBM, both regionally and globally.

## Supplementary Information

Supplementary Material 1.

## Data Availability

The data used in this research is available from the authors upon reasonable request.

## Abbreviations

DBM: Double burden of malnutrition
Food-EPI: Healthy Food Environment Policy Index
FOPL: Front-of-package label
INFORMAS: International Network for Food and Obesity/NCDs Research, Monitoring and Action Support
NCD: Non-communicable disease
SSB: Sugar-sweetened beverage
WASH: Water, sanitation, and hygiene
