# Supplementary material for: Policy implementation and recommendations to address the double burden of malnutrition in South Africa: expert assessment using the expanded Healthy Food Environment Policy Index (Food-EPI)
Source: BMC Med. 2025 Jul 1;23:360. doi: 10.1186/s12916-025-04191-y (PMC12220205; doi:10.1186/s12916-025-04191-y)
Supplement: Supplementary file 1 — Supplementary Material 1. [file 12916_2025_4191_MOESM1_ESM.docx]

| **Food-EPI South Africa 2024 - Prioritization of concrete actions** | | | |
| --- | --- | --- | --- |
|  | **Policy actions** |  |  |
|  | *Note: You* ***may assign up to 5 points per action*** *(split between importance and achievability). E.g., you could assign 3 points to importance and 2 points to achievability or 2 points to importance and 1 point to achievabilty for any given action.  In total,* ***you may assign a maximum of 22 points per criteria*** *(importance / achievability). The red colour indicates if you have assigned too many points in one category.* |  |  |
| **No** | **Action** | **Importance** | **Achievability** |
| P1 | Develop food composition standards and implement menu labelling for (quick service) restaurants. |  |  |
| P2 | Pass a legislation to regulate the promotion, sponsorship and advertisement of unhealthy food and drinks (with sugar, and other nutrients of concern (saturated fatty acids, salt)) in the school environment and other settings where children gather, enforceable with fines. |  |  |
| P3 | Enforce legislation to regulate the promotion, sponsorship, advertisement of unhealthy food and drinks (with sugar, and other nutrients of concern (saturated fatty acids, salt)) towards children in print and online/social media as well as other non-broadcast media, enforceable with fines. |  |  |
| P4 | Develop and implement policy to support restaurants in reducing food waste, including through donations of safe and healthy foods. |  |  |
| P5 | Implement strategies to increase the affordability of healthy foods with a focus on vulnerable populations (e.g., subsidies, removal of fiscal taxes). |  |  |
| P6 | Implement and/or increase taxes on unhealthy foods that will raise their price, in particular increase SSB taxation to the 20% threshold. |  |  |
| P7 | Implement income support programmes for healthy food. |  |  |
| P8 | Improve access to water, sanitation and hygiene in order to reduce food-borne diseases in public places. |  |  |
| P9 | Develop and implement zoning laws to limit unhealthy food outlets. |  |  |
| P10 | Develop and implement zoning laws to promote and enable outlets selling fruits and vegetables. |  |  |
| P11 | Develop and implement support systems for restaurants and food stores to promote healthy food. |  |  |
|  |  |  |  |
|  | **Overall Score** | 0 | 0 |
|  |  |  |  |
|  | Infrastructure and support actions |  |  |
|  | *Note: You* ***may assign up to 5 points per action*** *(split between importance and achievability). E.g., you could assign 3 points to importance and 2 points to achievability or 2 points to importance and 1 point to achievabilty for any given action.  In total,* ***you may assign a maximum of 12 points per criteria*** *(importance / achievability). The red colour indicates if you have assigned too many points in one category.* |  |  |
| **No** | **Action** | **Importance** | **Achievability** |
| I1 | Develop and implement policies to regulate relationships and influence of commercial industry on government. |  |  |
| I2 | Ensure implementation of evaluation of policies and programs on population nutrition in a timeous way to ensure accountability. |  |  |
| I3 | Allocate adequate funding for a population nutrition budget, government funded research, and the funding for health promoting agencies. |  |  |
| I4 | Develop and implement transparent and clearly-mandated platforms for interaction with both civil society and the commercial sector regarding issues of nutrition. |  |  |
| I5 | Strengthen cross-sectoral platforms for coordination of nutrition and nutrition-related policies and plans. |  |  |
| I6 | Include nutrition / health impact assessments as part of socio-economic impact assessment in food and agricultural policies. |  |  |
|  |  |  |  |
|  | **Overall Score** | 0 | 0 |
